# Non-invasive imaging of radiocesium dynamics in a living animal using a positron-emitting ^127^Cs tracer

**DOI:** 10.1038/s41598-020-73351-2

**Published:** 2020-10-15

**Authors:** Nobuo Suzui, Takuya Shibata, Yong-Gen Yin, Yoshihito Funaki, Keisuke Kurita, Hiroyuki Hoshina, Mitsutaka Yamaguchi, Shu Fujimaki, Noriaki Seko, Hiroshi Watabe, Naoki Kawachi

**Affiliations:** 1 National Institutes for Quantum and Radiological Science and Technology (QST), Takasaki Advanced Radiation Research Institute, Gunma, 370-1292 Japan; 2 Japan Atomic Energy Agency, Quantum Beam Science Center, Gunma, 370-1292 Japan; 3 Tohoku University, Cyclotron and Radioisotope Center (CYRIC), Miyagi, 980-8578 Japan; 4 Tohoku University, Graduate School of Biomedical Engineering, Miyagi, 980-8579 Japan; 5 Present Address: Japan Atomic Energy Agency, Collaborative Laboratories for Advanced Decommissioning Science (CLADS), Fukushima, 979-1151 Japan; 6 Present Address: Japan Atomic Energy Agency, Materials Sciences Research Center, Ibaraki, 319-1195 Japan; 7 Present Address: National Institutes for Quantum and Radiological Science and Technology (QST), Institute for Quantum Life Science, Chiba, 263-8555 Japan

**Keywords:** Positron-emission tomography, Environmental impact

## Abstract

Visualizing the dynamics of cesium (Cs) is desirable to understand the impact of radiocesium when accidentally ingested or inhaled by humans. However, visualization of radiocesium in vivo is currently limited to plants. Herein, we describe a method for the production and purification of ^127^Cs and its use in visualizing Cs dynamics in a living animal. The positron-emitting nuclide ^127^Cs was produced using the ^127^I (α, 4n) ^127^Cs reaction, which was induced by irradiation of sodium iodide with a ^4^He^2+^ beam from a cyclotron. We excluded sodium ions by using a material that specifically adsorbs Cs as a purification column and successfully eluted ^127^Cs by flowing a solution of ammonium sulfate into the column. We injected the purified ^127^Cs tracer solution into living rats and the dynamics of Cs were visualized using positron emission tomography; the distributional images showed the same tendency as the results of previous studies using disruptive methods. Thus, this method is useful for the non-invasive investigation of radiocesium in a living animal.

## Introduction

The accident at Tokyo Electric Power Company’s Fukushima Daiichi Nuclear Power Station in March 2011 caused various radioactive materials to fall over a wide area^1^. In particular, ^137^Cs, which has a half-life of 30 years, had a serious impact on the affected area^2,3^. Subsequently, interest in the dynamics of radiocesium in animals, plants, and humans has increased. There are concerns about where in the body radiocesium ingested by humans accumulates, and how quickly it is excreted.

To more accurately calculate the internal exposure dose of radiocesium, it is necessary to analyze the dynamics of radiocesium in animals in more detail. The current calculation of internal exposure doses of radiocesium is based on data from animal studies, and human urine and feces. More than 90% of ingested soluble cesium (Cs) is absorbed in the gastrointestinal tract^4^, but the real-time behavior and kinetics immediately after ingestion remain unclear. For example, spherical Cs-bearing particles (Cs-balls) were emitted in the Fukushima nuclear accident^5^, and the dynamics of such Cs-balls in the human body are unknown.

Visualizing the dynamics of radiocesium in vivo is a powerful method for studying the dynamics of radiocesium. In the field of plant science, there have been reports of visualizing the dynamics of radiocesium in living plants. Kobayashi et al. have succeeded in visualizing the β rays of ^137^Cs using a real-time radioisotope imaging system^6^. Kurita et al. have succeeded in visualizing the dynamics of ^137^Cs by imaging Cherenkov light emitted from β rays with an ultra-sensitive CCD camera^7^. Although these methods are suitable for the visualization of radiocesium in plants with a small tissue thickness, they cannot visualize the dynamics of radiocesium in animals, which have a large tissue thickness. Kawachi et al. successfully visualized the 661 keV γ rays of ^137^Cs with a pinhole-type gamma camera^8^. However, this technique provides two-dimensional images, which are sufficient for plant research but are not suitable for animal research.

Positron imaging is widely used to non-destructively visualize the dynamics of positron-emitting radionuclides in vivo. The most common use of positron imaging is cancer screening by positron emission tomography (PET), which exploits the tendency of fluorodeoxyglucose labeled with the positron-emitting radionuclide ^18^F to accumulate in cancer cells. Additionally, PET has been used in medical research to analyze the kinetics of drugs labeled with ^11^C, ^13^N, and ^15^O. As for the positron-emitting nuclide of Cs, McKee et al. reported that ^132^Cs (half-life of 6.48 d, β^+^ intensity of 0.43%) was produced and administered to animals to verify the applicability of ^132^Cs as a tumor marker^9^. However, because ^132^Cs was produced by irradiating stable Cs, ^132^Cs could not be purified, resulting in low specific activity. Therefore, a ^132^Cs tracer is not suitable for non-invasive imaging of radiocesium in vivo where the tracer must exhibit high specific activity.

In this study, the positron-emitting nuclide ^127^Cs (half-life of 6.25 h, β^+^ intensity of 3.04%) without stable Cs was produced by irradiating iodine with a helium ion beam accelerated by a cyclotron. Then, we developed a unique purification method to adsorb and elute only ^127^Cs using a material that specifically collects Cs. Additionally, by administering the purified ^127^Cs tracer to living animals, we verified that ^127^Cs is useful for visualization of the short-term dynamics of radiocesium.

## Results and discussion

### Production of ^127^Cs

^127^Cs was produced by the ^127^I (α, 4n)^127^Cs reaction. A sodium iodide (NaI) target plate was irradiated with a ^4^He^2+^ beam from an AVF cyclotron at Takasaki Ion Accelerators for Advanced Radiation Application (TIARA). The incident energy on the target was set to 55 MeV with reference to the cross-section from the experimental data of Sathik et al.^10^ and data from the ACSELAM library^11^ (Supplementary Fig. 1). Under these irradiation conditions, ^129^Cs was also produced by the ^127^I (α, 2n)^129^Cs reaction. Supplemental Table 1 lists nuclides that were potentially generated.

**Figure 1:**
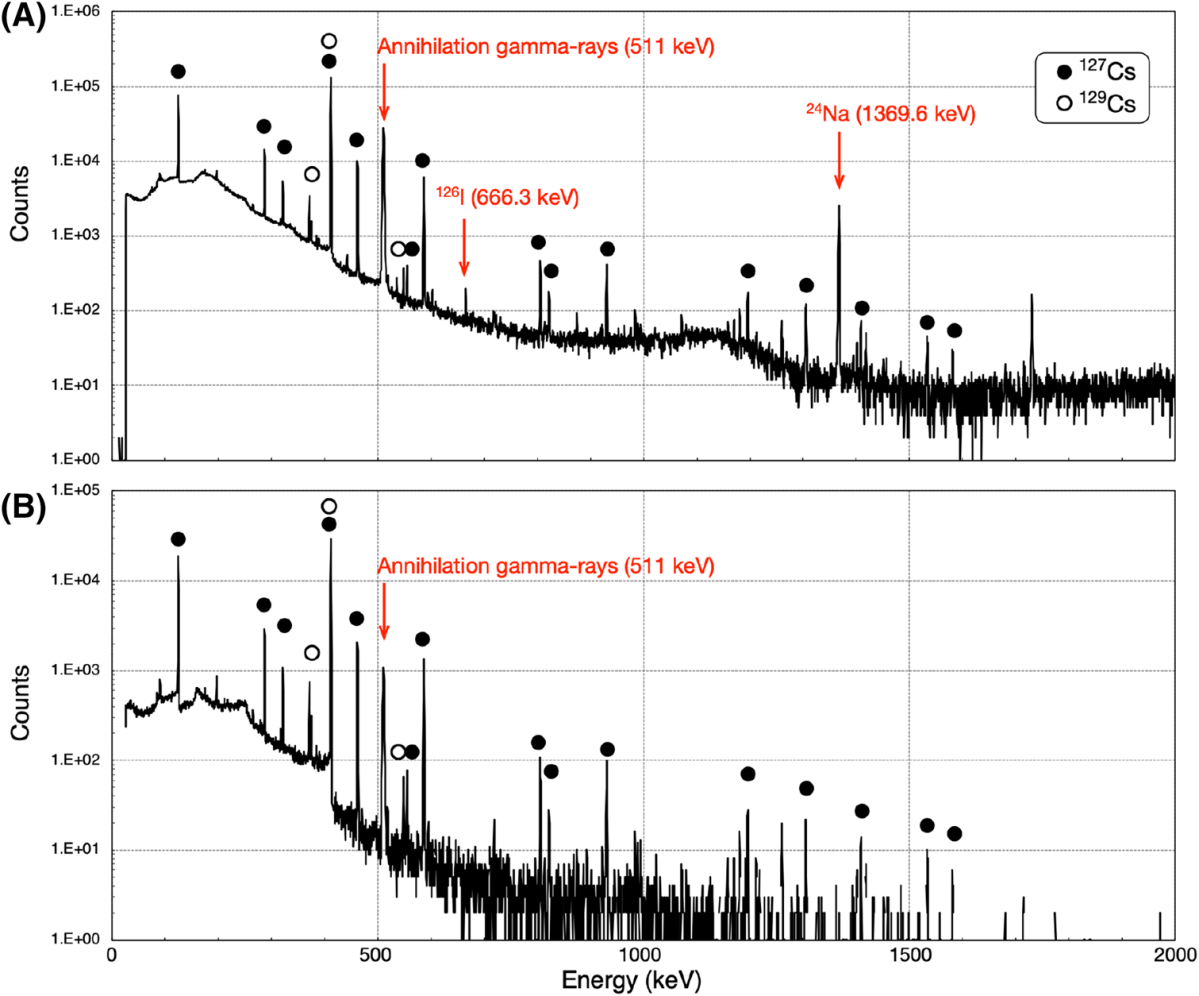
Gamma spectrum of the mother and purified solutions. (**A**) Gamma-ray spectrum of the mother solution containing a hundredth (10 µL) of the total volume (1 mL), which was measured for 20 min at 21.5 cm from the detector, 47 min after the end of irradiation. (**B**) Gamma-ray spectrum of the purified solution containing a thousandth (10 μL) of the total volume (10 mL), which was measured for 20 min at 3.5 cm from the detector, 124 min after the end of irradiation.

Figure 1A shows the gamma spectrum of the mother solution, in which the irradiated NaI target was dissolved. Under the irradiation conditions in this study, the production yields of ^127^Cs and ^129^Cs at the end of irradiation were 80.1 ± 6.5 and 1.3 ± 0.04 MBq/µAh, respectively (n = 6). Additionally, ^24^Na, ^124^I, and ^126^I were also produced as by-products, which could be used effectively as radioactive tracers for sodium and iodide ions from the NaI target in the subsequent purification experiments. The decay data from the radionuclides produced in this study are summarized in Supplementaary Table 2. In the gamma-ray spectrum, the other peaks could not be identified, with the exception of ^127^Xe (daughter nuclide of ^127^Cs) among the nuclides listed in Supplementary Table 1.

### Purification of ^127^Cs tracer

**Figure 2:**
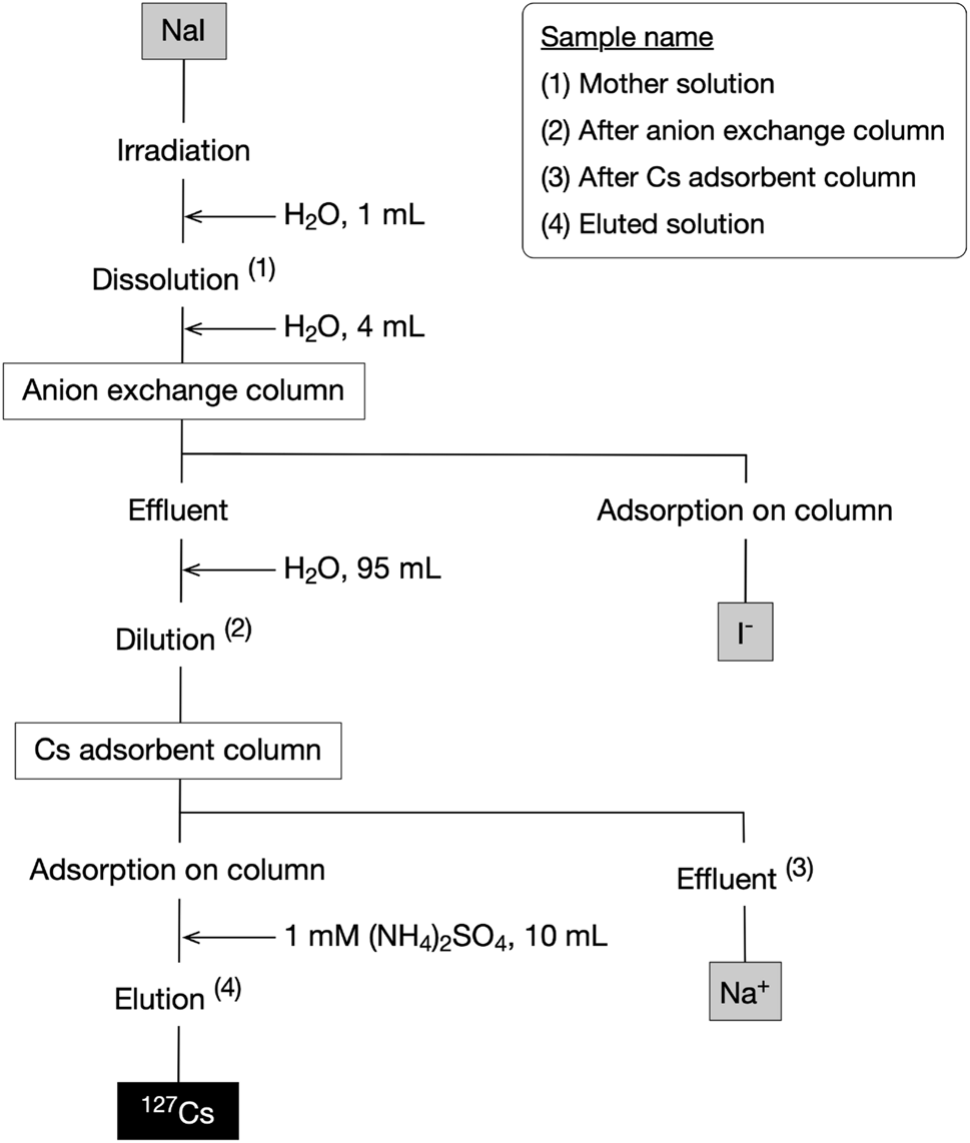
Procedure for the separation of ^127^Cs from the irradiated NaI target. The numbers (1)–(4) indicate the sampling points (consistent with Table 1) in the process of purification.

The procedure for the separation of ^127^Cs from the irradiated NaI target is shown schematically in Fig. 2. Table 1 shows the radioactivity of the nuclides in each purification step when the irradiation condition was 0.5 µA × 2 h. First, we loaded the mother solution on to an anion-exchange column. The results showed that ^124^I and ^126^I were not detected in the effluent solution from the anion-exchange column, which indicated that iodide ions derived from the NaI target were removed.

**Table 1:** Radioactivity of the nuclides at each purification step.

Sample name	^127^Cs (MBq)	^129^Cs (MBq)	^124^I (kBq)	^126^I (kBq)	^24^Na (MBq)
(1) Mother solution	80.1 (6.5)	1.3 (0.04)	124.9 (21.8)	114.1 (14.8)	2.6 (0.2)
(2) After anion-exchange column	70.9 (10.5)	1.1 (0.09)	ND	ND	2.4 (0.4)
(3) After Cs adsorbent column	1.5 (1.2)	ND	ND	ND	2.5 (0.3)
(4) Eluted solution	37.4 (5.6)	0.6 (0.12)	ND	ND	ND

All radioactivity values are corrected for decay at the end of irradiation. Each value represents the mean (SD) for six experiments.

*ND* not detected.

Next, to separate ^127^Cs from sodium ions, we used fibrous Cs adsorbent, which was synthesized by radiation-induced graft polymerization to introduce ammonium 12-molybdophosphate (AMP) as an adsorption ligand onto a polyethylene trunk fabric. This material has been reported to adsorb Cs specifically^12^. We considered that this adsorbent is effective in purifying trace amounts of ^127^Cs under the extreme condition in this study, where the Na^+^/^127^Cs ratio was 0.8 mmol/4.3 pmol (80 MBq), a 10^8^-fold molar number difference. For efficient adsorption of ^127^Cs on the Cs adsorbent column, we reduced the concentration of the sodium ions in the effluent solution by increasing the volume of the solution from 5 to 100 mL. We then passed the solution through the Cs adsorbent column. The results showed that 98% ± 2% (n = 6) of ^127^Cs was adsorbed on the Cs adsorbent packed into the column (Table 1). After washing the column with 100 mL of H_2_O, we finally passed 10 mL of 1 mM (NH_4_)_2_SO_4_ through the Cs adsorbent column. The results showed that 55% ± 4% (n = 6) of ^127^Cs was eluted from the adsorbent but ^24^Na was not detected in the eluted solution (Table 1), which indicated that sodium ions derived from the NaI target were removed. Figure 1B shows the gamma spectrum of the eluted solution, which also indicated that the peaks of ^24^Na and ^126^I had disappeared in the final solution. Supplementary Fig. 2 shows the gamma-ray spectrum of the mother solution and the final ^127^Cs tracer solution measured for 3 h. Because the detection limits of the Ge detector for ^124^I and ^126^I under these conditions are 3.39 and 2.58 Bq, respectively, the maximum estimated radioactivities in the final ^127^Cs tracer solution at the end of irradiation are less than 5.1 and 3.8 kBq, respectively. Therefore, we calculated that even at the three half-lives of ^127^Cs (18.75 h), the ratios of the positrons from ^124^ and ^126^I to those from ^127^Cs are maximally less than 0.68% and 0.03%, respectively. Because this study was performed by the no-carrier-added production method, the specific activity of ^127^Cs was calculated as 1.5 × 10^17^ Bq/g, which is much higher than the 1.0 × 10^6^ Bq/g (28 µCi/g) calculated for the previous ^132^Cs report^9^.

**Figure 3:**
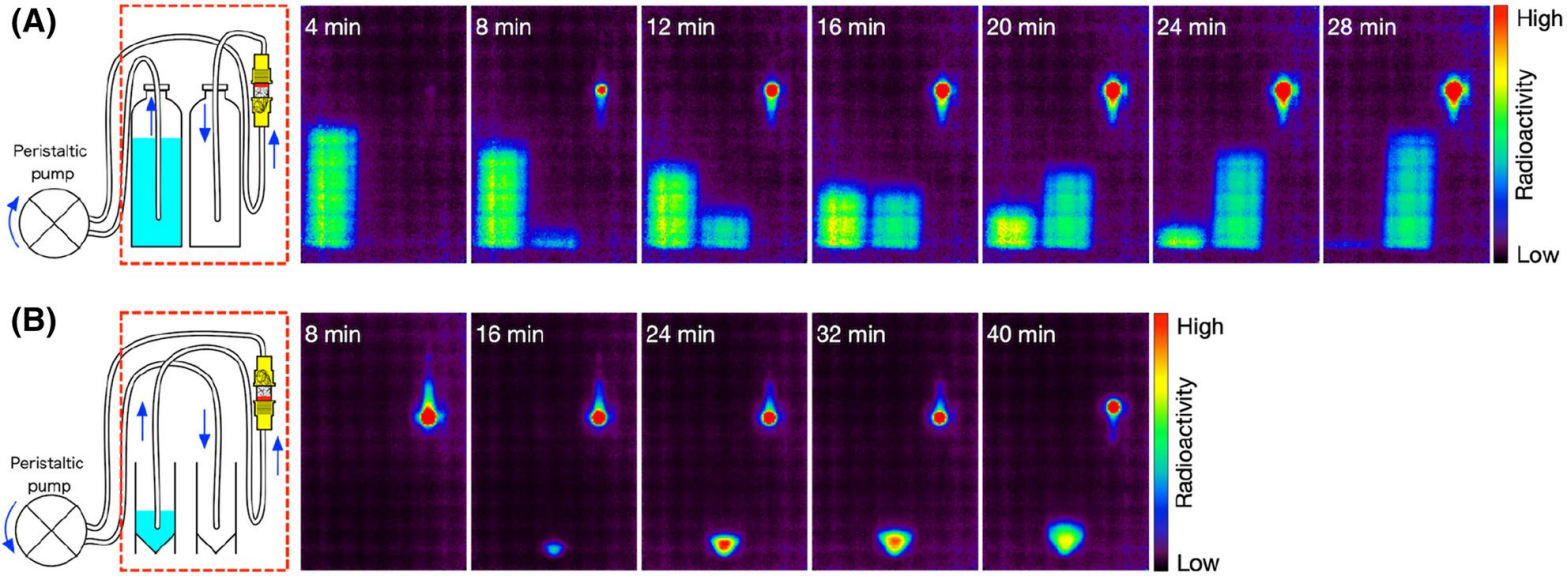
Serial images of the purification step using the Cs adsorbent column. (**A**) The adsorbing step of ^127^Cs to the Cs adsorbent column. Each frame is an image integrated every 4 min. (**B**) The eluting step of ^127^Cs from the Cs adsorbent column. Each frame is an image integrated every 8 min. The red-dashed rectangles and the blue arrows in the left illustration indicate the field of view of the PPIS, and the direction of the solution flow, respectively.

We simultaneously visualized the movement of the positron-emitting nuclides in the purification step of the Cs adsorbent column using a planar positron imaging system (PPIS). Figure 3A shows the process of ^127^Cs adsorption on the column. The effluent solution of the anion-exchange column diluted to 100 mL was flowed from the left bottle to the right bottle via the Cs adsorbent column in the direction from the bottom to the top over 30 min. We observed that the signal at the Cs adsorbent column position gradually increased. However, signals were also seen in the right bottle, even though the effluent solution from the Cs adsorbent column contained little radioactivity from ^127^Cs (Table 1). The half-life of the signal of the right bottle was measured with the planar positron imaging system and was estimated to be 109.9 min (Supplementary Fig. 3). It has been reported that ^18^F is produced by the ^23^Na (α, n + 2α)^18^F reaction when sodium is irradiated with a ^4^He^2+^ beam^13^. The calculated cross-section of ^18^F at 55 MeV was 21.7 m barn (Supplementary Table 1) and the radioactivity of ^18^F quantified from the data in Supplementary Fig. 3 was 2.2 MBq. Thus, we believe that the effluent solution from the Cs adsorbent column may contain ^18^F, which has a half-life of 109.8 min. The estimated value (109.9 min) of the half-life of the signal of the right bottle was slightly longer than the half-life of ^18^F and this was assumed to be because a small amount of ^127^Cs was included in the effluent solution from the Cs adsorbent column (Table 1). Because the affinity for fluorine of the anion exchange column used in this study is significantly lower than the affinity for iodine, it is possible that ^18^F could pass through the column.

Figure 3B shows the process of ^127^Cs elution from the Cs adsorbent column. First, to minimize re-adsorption of ^127^Cs to the Cs adsorbent, the column was turned upside down. Then, 10 mL of 1 mM (NH_4_)_2_SO_4_ was flowed from the left vial to the right vial via the column in the direction from the bottom to the top over 30 min. In the last 10 min, the column was turned upside down and the solution in the column was pushed out with air.

**Figure 4:**
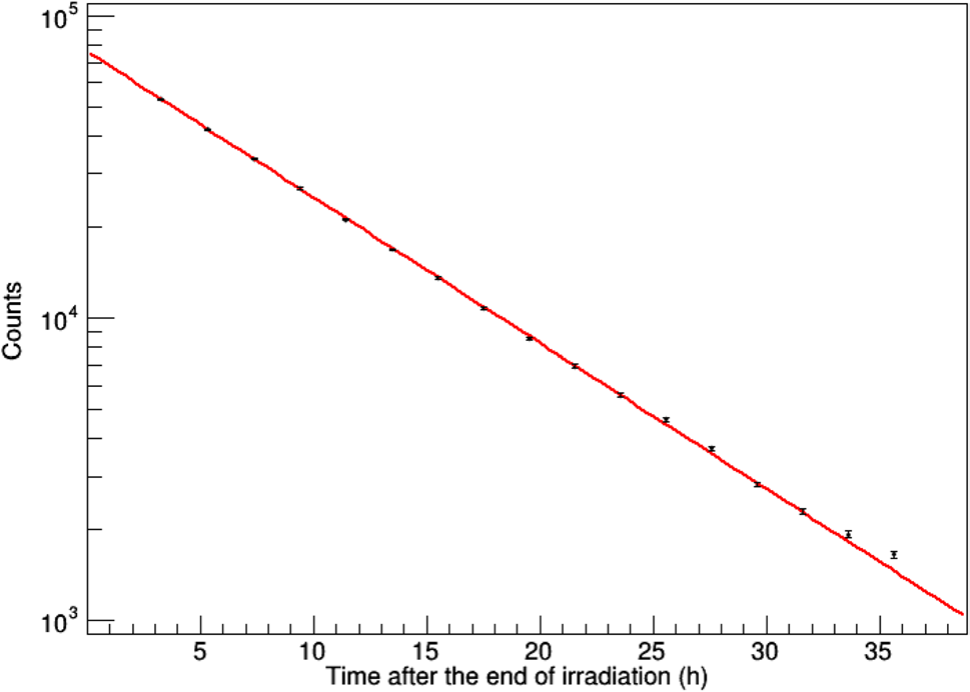
Decay of the peak count of annihilation gamma rays in the final ^127^Cs tracer solution. Ten microliters of the solution were measured for every 2 h at 7.0 cm from the detector. The red line represents the result of one-component fitting. Error bars represent statistical errors in measurement.

To verify the contamination of ^18^F into the final ^127^Cs tracer solution, we calculated the decay time of the peak counts of annihilation gamma ray (511 keV) with the data obtained by measuring the final solution every 2 h with the Ge detector (Fig. 4). By one-component fitting, we calculated that the half-life of the peak count was 6.279 ± 0.015 h, which is almost consistent with the half-life of ^127^Cs. Furthermore, using two-component fitting (^127^Cs and ^18^F) of this decay curve, the percentage of the ^18^F component in this peak count at the start time was calculated as 0.663 ± 1.273%. These results indicate that the contamination of ^18^F into the final ^127^Cs tracer solution was extremely low. Additionally, the estimate indicates that the radionuclidic purity of ^127^Cs in the 36 h after the end of irradiation was more than 99%.

The principle of Cs adsorption and elution from the Cs adsorbent can be described as follows: Cs is adsorbed by a substitution reaction with ammonia from the AMP in the column structure, and then the adsorbed Cs is replaced by ammonia from contact with the ammonium solution.

**Figure 5:**
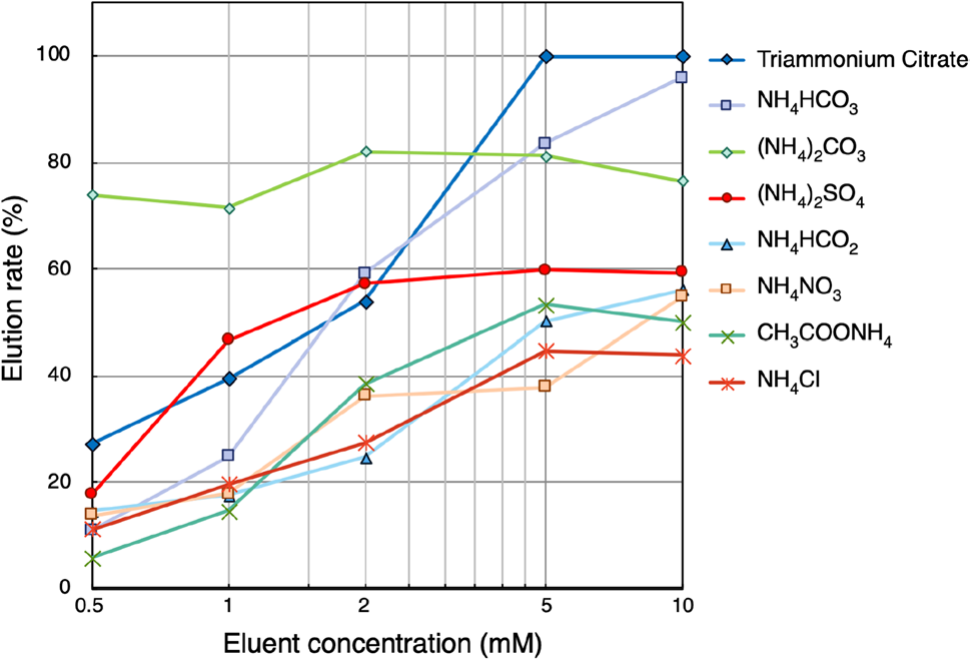
Elution rate of Cs from the adsorbent using various ammonium compounds.

Figure 5 shows the elution rates of Cs from the adsorbent with various concentrations of different ammonium compounds in a batch experiment using non-radioactive Cs. We selected an eluate solution using two criteria to make the final tracer solution suitable for physiological study for both animal and tracer experiments in general biology, including plant experiments: the ion concentration should be as low as possible, and the pH should be close to neutral. Also, it was desired that the elution rate be at least 50% or more for efficient purification. When the ion concentration was limited to 1 mM, (NH_4_)_2_CO_3_ and (NH_4_)_2_SO_4_ were identified as candidates. The elution rate of 1 mM (NH_4_)_2_CO_3_ was higher than that of (NH_4_)_2_SO_4_, but the pH values were 9.2 and 5.7, respectively. Therefore, we used a solution of 1 mM (NH_4_)_2_SO_4_, for eluting ^127^Cs from the Cs absorbent column in the purification experiment.

To verify the desorption of AMP particles from the adsorbent, the molybdenum concentration of the solution eluted with 1 mM ammonium sulfate was measured and the desorption rate was calculated as 0.0086%. This indicates that the possibility of contamination of AMP particle into the final tracer solution is quite low when this adsorbent is used for purification.

### PET imaging

**Figure 6:**
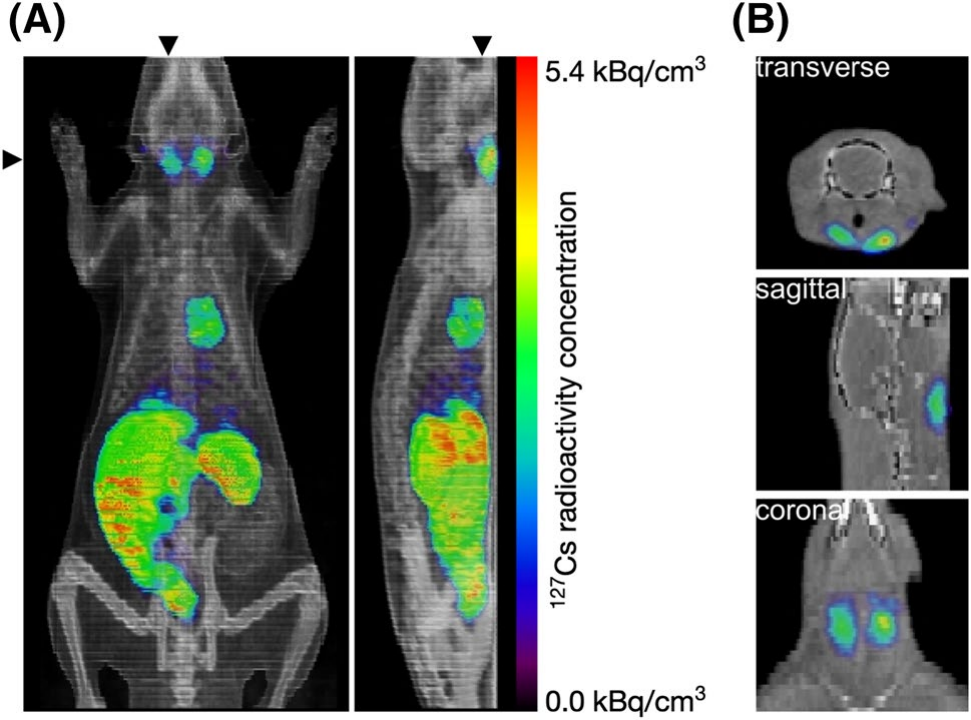
Integrated image of PET imaging of ^127^Cs in a living rat. (**A**) Four-hour integrated image of ^127^Cs in the whole body of the rat in Experiment 2. The triangles indicate the location of the cross-section. (**B**) Cross-sectional view of the integrated image of ^127^Cs in the neck.

The purified ^127^Cs tracer solution was injected into the tail vein of a living rat, and the dynamics of Cs were visualized using PET, in duplicate. The doses of ^127^Cs for Experiments 1 and 2 were 2 and 11 MBq, and the imaging times were 3 and 4 h, respectively. To make the dose as low as reasonably achievable, we administered 2 MBq of ^127^Cs in Experiment 1. To obtain more clear PET images, we administered 11 MBq of ^127^Cs in Experiment 2, which we consider to be the more desirable dose. Figure 6A shows a 4-h integrated image of ^127^Cs in the whole body of the rat in Experiment 2. This result indicated that ^127^Cs passed or accumulated in the neck, heart, small intestine, and kidney. Figure 6B shows a cross-sectional view of the integrated image of ^127^Cs in the neck. From comparison with the CT image, the signal for ^127^Cs was found to be localized in the salivary glands in the neck. The localization pattern of the ^127^Cs signal was the same in Experiment 1 (Supplementary Fig. 4).

**Figure 7:**
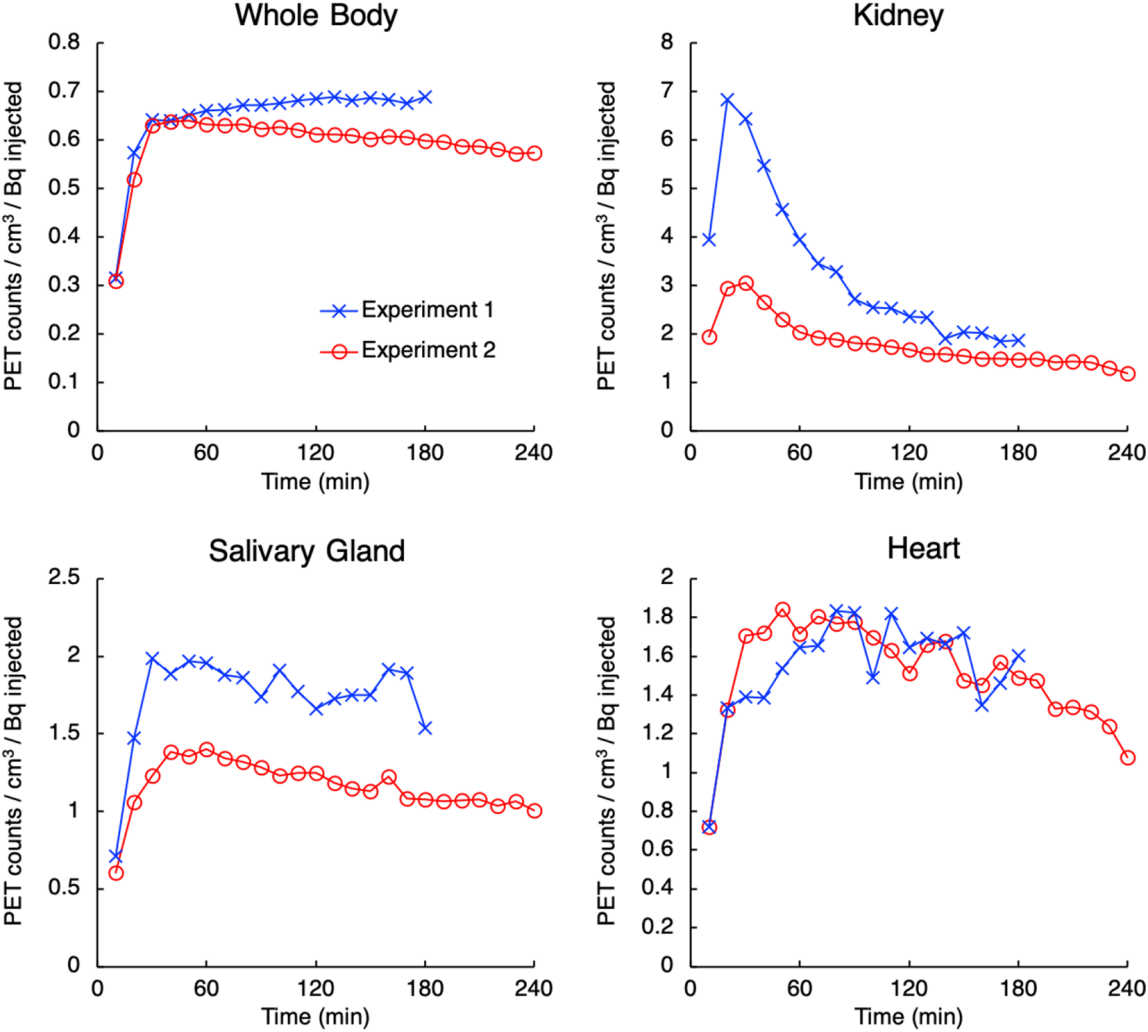
Time course for ^127^Cs counts in the whole body and selected organs.

Figure 7 shows the time course of ^127^Cs PET counts in the whole body and in each organ. All counts were decay corrected at the start time of PET imaging and normalized to the dose of ^127^Cs and the volume of each region. Although there were differences in the normalized PET counts in the two experiments, the same trend was observed over each time course. The time course of the whole body indicated that the ^127^Cs tracer injected into the tail vein moved into the field of view of the PET in 30 min. The accumulation of ^127^Cs in the kidney reached a peak at 20–30 min and was rapidly excreted. In the salivary glands and heart, the accumulation of ^127^Cs reached a peak at 30–60 min and was slowly excreted. In contrast, although the molecular number of ^127^Cs administered in Experiments 1 and 2 differed five-fold, the kinetics of ^127^Cs exhibited the same trend and no saturation was observed even with the five-fold difference in molecular number, which reflects no region of specific affinity of ^127^Cs in rats. Moreover, our method can produce no-carrier-added ^127^Cs and no influence of injected radioactivity is expected.

Kaikkonen et al*.* have measured the radioactivity using a gamma counter in different tissues of goats 30 min after administrating ^134^Cs intravenously^14^. The results showed that the highest relative concentration of ^134^Cs was found in the kidney cortex, followed by the salivary gland, cardiac muscle, and small intestine. Sasaki et al*.* have reported that cattle fed with ^134^Cs and ^137^Cs for several weeks had more radiocesium accumulation in the kidney than in other organs^15^. Although the tested animal species and methods of radiocesium administration are different, these studies are consistent with the results of our study, which suggests that ^127^Cs can be used to correctly visualize Cs dynamics in the living animal body.

To the best of our knowledge, this is the first study to visualize the dynamics of radiocesium in living animals. In this study, we administered ^127^Cs to animals by intravenous injection. In the future, it should be possible to non-invasively calculate the transfer rate of radiocesium to each organ by administering ^127^Cs through ingestion or inhalation. The calculated results will contribute to the construction of more accurate models of Cs dynamics and improvements in the accuracy of effective dose coefficients. Furthermore, because of the short half-life of ^127^Cs (6.25 h), repeated experiments using the same animal are possible. For example, the effects of various types of internal decontamination agents could be evaluated accurately.

## Methods

### Production of ^127^Cs

^127^Cs was produced by the ^127^I (α, 4n)^127^Cs reaction. A target plate with a diameter of 10 mm and a thickness of 0.5 mm was made by pressing 125 mg of NaI reagent (Fujifilm Wako Pure Chemicals, Tokyo, Japan). The NaI target plate was irradiated with a ^4^He^2+^ beam for 2–3.25 h, at a beam current of 0.5 µA and an incident energy of 55 MeV, delivered from an AVF cyclotron at TIARA.

### Column purification of ^127^Cs tracer

After the irradiation, the NaI target was dissolved in 1 mL of H_2_O. To remove iodide ions, the solution was loaded onto an anion-exchange column (Poly-Prep #731-6211; Bio-Rad, Hercules, CA, USA) and an additional 4 mL of H_2_O was loaded.

To separate ^127^Cs from the sodium ions, we used a fibrous Cs adsorbent (KURANGRAFT-Cs, Kurashiki Textile Manufacturing Co. Ltd., Kurashiki, Japan), which comprised fibrous polyethylene trunk material cross-linked with a Cs adsorption ligand, ammonium 12-molybdophosphate (AMP). Supplementary Fig. 5 shows the structure of the Cs adsorbent column. The fibrous Cs adsorbent was cut out into 0.7-cm-diameter disks, and 40 disks were packed into a glass chromatography column (Econo-Column Chromatography Columns #737-0707, Bio-Rad) with quartz wool. The volume of Cs adsorbent in the column was 1.0 cm^3^ (0.7-cm diameter × 2.5-cm height). This glass chromatography column was used as the Cs adsorbent column. The effluent solution of the anion-exchange column was diluted to 100 mL with H_2_O and passed through the Cs adsorbent column at a flow rate of approximately 4 mL/min using a peristaltic pump (Model: 13-876-2; Thermo Fisher Scientific, Waltham, MA, USA). The Cs adsorbent column was washed by passing 100 mL of H_2_O at the same flow rate, and then ^127^Cs was eluted by passing 10 mL of 1 mM (NH_4_)_2_SO_4_ (Fujifilm Wako Pure Chemicals) through the column at a flow rate of approximately 0.4 mL/min using a peristaltic pump (Model: 13-876-1; Thermo Fisher Scientific). The purification process using the Cs adsorbent column was imaged using a planar positron imaging system (PPIS-4800; Hamamatsu Photonics, Hamamatsu, Japan).

### Radiochemical analysis

The produced radionuclides were characterized by a HPGe detector (crystal diameter of 58 mm and length of 67.3 mm) connected to a multichannel analyzer (SEIKO 7800 MCA). The radioactivity was determined by considering the γ-ray energy at 462.3 (^127^Cs), 371.9 (^129^Cs), 1369.6 (^24^Na), 602.7 (^124^I), and 666.3 keV (^126^I).

### Decay fitting analysis

The half-life of the radioactivity in the effluent solution (Supplementary Fig. 3) and the eluted solution (Fig. 4) from the Cs adsorbent column was estimated by fitting the data with the following one-component formula:

$$ N = A \times 2^{{ - \frac{{\left( {t - t_{0} } \right)}}{\tau }}} , $$

where $t$ and $N$ represent the measurement time and the count, respectively. The start time of the measurement and the count at the start time are represented by $t_{0}$ and $A$, respectively. The half-life is represented by $\tau$. Two parameters, $A$ and $\tau$, were estimated by fitting the data ($t$ and $N$) with the formula with fixing $t_{0}$ at the experimental value of the start time.

The effect of the ^18^F contamination in the eluted solution from the Cs adsorbent was also evaluated, by fitting the data with the following two-component formula:

$$ N = A_{{{\text{Cs}}}} \times 2^{{ - \frac{{\left( {t - t_{0} } \right)}}{{\tau_{{{\text{Cs}}}} }}}} + A_{{\text{F}}} \times 2^{{ - \frac{{\left( {t - t_{0} } \right)}}{{\tau_{{\text{F}}} }}}} , $$

where $A_{{{\text{Cs}}}}$ and $A_{{\text{F}}}$ and $\tau_{{{\text{Cs}}}}$ and $\tau_{{\text{F}}}$ represent the counts at the start time and the half-lives for Cs and F, respectively. Two parameters, $A_{{{\text{Cs}}}}$ and $A_{{\text{F}}}$, were estimated by fitting the data with the formula by fixing $\tau_{{{\text{Cs}}}}$ and $\tau_{{\text{F}}}$ at 6.25 h and 109.8 min, respectively, and then fixing the start time $t_{0}$ at 0.

### Cs elution rate analysis

The Cs-adsorbed adsorbents were prepared as follows. The fibrous Cs adsorbent was cut into 0.5-cm-diameter disk (8.3 mg). The adsorbent disk was placed in 10 mL of stable Cs solution with a concentration of 7.5 nM (1 ppb). After shaking for 4 h at 25 °C, the Cs adsorbent disk was removed from the Cs solution and the concentration of the remaining Cs was measured using inductively coupled plasma-mass spectrometry (7700 × ICP-MS, Agilent Technologies, Santa Clara, CA, USA). The calculated adsorption rate of Cs to the adsorbent was 84% ± 7% (n = 40).

The elution was carried out with aqueous solutions of (NH_4_)_3_C_6_H_5_O_7_, NH_4_HCO_3_, (NH_4_)_2_CO_3_, (NH_4_)_2_SO_4_, NH_4_HCO_2_, NH_4_NO_3_, CH_3_COONH_4_, and NH_4_Cl for the batch test. The concentrations of the ammonium compound solutions were adjusted to 0.5, 1.0, 2.0, 5.0, and 10 mM. The adsorbent disk was soaked in 5 mL of aqueous solutions of each ammonium compound, and was continuously shaken for 20 h at 25 °C. After elution, the concentration of Cs in the ammonium solutions was measured using ICP-MS. The elution rate of Cs was calculated by the following equation:

$$ {\text{Elution}}\,{\text{rate}}\, ({\text{\% }}) = \left( {C_{E} /C_{A} } \right) \times 100, $$

where *C*_*A*_ and *C*_*E*_ are the amount of adsorbed Cs in the adsorbent and eluted Cs in the ammonium solutions, respectively.

### PET imaging

PET imaging experiments using two living rats were performed (Experiments 1 and 2). The eluted solution containing ^127^Cs was evaporated to dryness in a glass vial and transported from TIARA to Tohoku University. Approximately 300 µL of physiological saline solution (154 mM NaCl) was added to the glass vial, and the ^127^Cs was recovered. The final concentration of (NH_4_)_2_SO_4_ was 33 mM. A tracer solution containing 2 or 11 MBq of ^127^Cs was injected to a 6-week-old male Wistar rats (132 or 136 g) via tail vein under inhalation anesthesia with 2% isoflurane in air. The distribution of ^127^Cs in the living rat was imaged using a small-animal PET instrument (Clairvivo PET^16^; Shimadzu, Japan) for 3 or 4 h. The tracer was injected 15.3 or 14.2 h after the end of irradiation. A list-mode DRAMA algorithm^17^ was used for image reconstruction with an iteration of 1 and sub-iterations of 100. The PET image had a matrix size of 128 × 128 × 213 and each voxel size was 0.7 mm^3^. No attenuation correction was performed, but we did conduct scatter correction using emission scatter convolution subtraction^18^. ^18^F solution was used for scanner calibration. The energy window for the scanner was set to 250–750 keV. A CT scan of the rat was also performed using a small-animal CT instrument (Clairvivo CT; Shimadzu) before the injection of the ^127^Cs tracer solution. Quantitative analysis of the PET images was performed with Amide software^19^.

### Ethics approval

All experimental procedures conformed to "Regulations for Animal Experiments and Related Activities at Tohoku University", and were reviewed by the Institutional Laboratory Animal Care and Use Committee of Tohoku University, and finally approved by the President of University. Ethical approval in this study has been obtained from the Institutional Laboratory Animal Care and Use Committee of Tohoku University.

## Supplementary information

Supplementary Information.
