# Supplementary material for: Non-invasive imaging of radiocesium dynamics in a living animal using a positron-emitting 127Cs tracer
Source: Sci Rep. 2020 Oct 15;10:16155. doi: 10.1038/s41598-020-73351-2 (PMC7567787; doi:10.1038/s41598-020-73351-2)
Supplement: Supplementary file 1 — Supplementary Information. [file 41598_2020_73351_MOESM1_ESM.docx]

**Full title:**

Non-invasive imaging of radiocesium dynamics in a living animal using a positron-emitting ^127^Cs tracer

Nobuo Suzui^1,^*, Takuya Shibata^2,^†, Yong-Gen Yin^1^, Yoshihito Funaki^3^, Keisuke Kurita^1,^†, Hiroyuki Hoshina^1^, Mitsutaka Yamaguchi^1^, Shu Fujimaki^1,^†, Noriaki Seko^1^, Hiroshi Watabe^1,3,4^, and Naoki Kawachi^1^

^1^National Institutes for Quantum and Radiological Science and Technology (QST), Takasaki Advanced Radiation Research Institute, Gunma 370-1292, Japan

^2^Japan Atomic Energy Agency, Quantum Beam Science Center, Gunma 370-1292, Japan

^3^Tohoku University, Cyclotron and Radioisotope Center (CYRIC), Miyagi 980-8578, Japan

^4^Tohoku University, Graduate School of Biomedical Engineering, Miyagi 980-8579, Japan

†Present address: Japan Atomic Energy Agency, Collaborative Laboratories for Advanced Decommissioning Science (CLADS), Fukushima 979-1151, Japan (T.S.); Japan Atomic Energy Agency, Materials Sciences Research Center, Ibaraki 319-1195, Japan (K.K.); National Institutes for Quantum and Radiological Science and Technology (QST), Institute for Quantum Life Science, Chiba 263-8555, Japan (S.F.)

*Corresponding author: suzui.nobuo@qst.go.jp


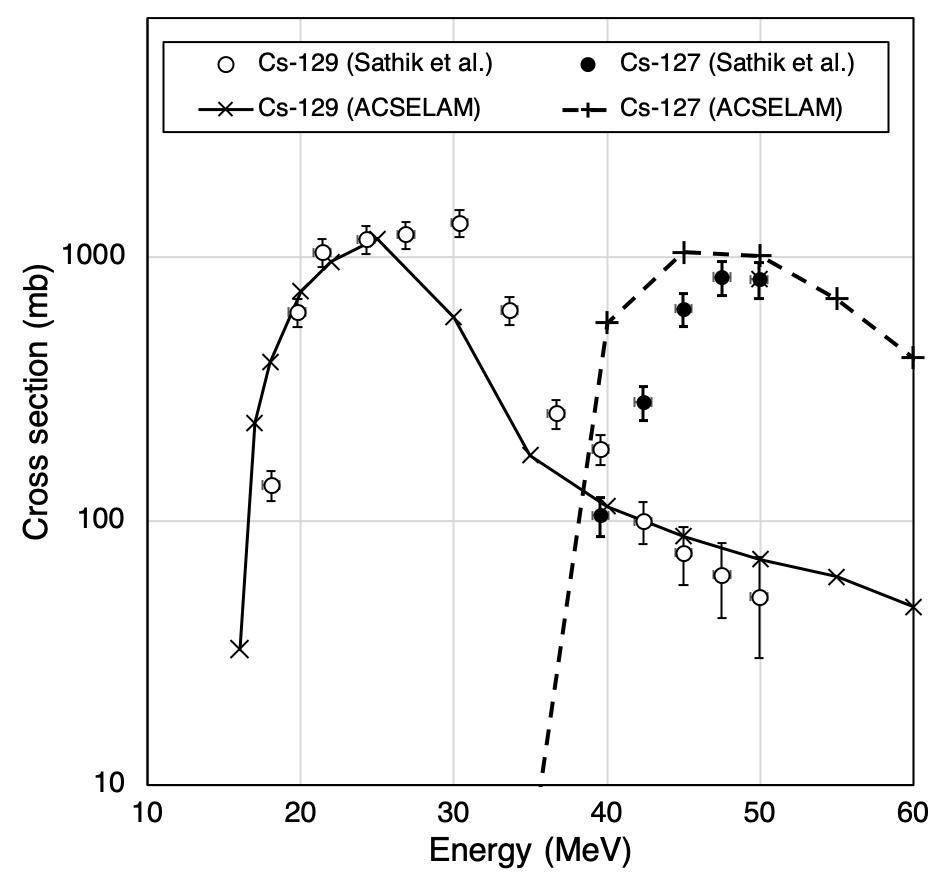


**Supplemental Figure 1.** Cross-sections for the reactions of ^127^I (α, 4n)^127^Cs and ^127^I (α, 2n)^129^Cs from the experimental data of Sathik *et al*. and data from the ACSELAM library.

**Supplemental Table 1.** Nuclides potentially generated in this study.

| Nuclide | Calculated cross section (m barn) |
| --- | --- |
| ^127^Cs | 6.95E+02 |
| ^126^Cs | 3.76E+02 |
| ^127^Xe | 2.30E+02 |
| ^128^Cs | 1.65E+02 |
| ^129^Cs | 6.15E+01 |
| ^124^I | 4.52E+01 |
| ^24^Na | 4.48E+01 |
| ^22^Na | 3.49E+01 |
| ^18^F | 2.17E+01 |
| ^25^Na | 1.44E+01 |
| ^21^Na | 1.17E+01 |
| ^128^I | 7.09E+00 |
| ^125^I | 7.03E+00 |
| ^15^O | 5.97E+00 |
| ^129^I | 5.83E+00 |
| ^125^Xe | 5.83E+00 |
| ^130^Cs | 5.20E+00 |
| ^126^I | 1.79E+00 |

Radionuclides with a cross-section exceeding 1 m barn at 55 MeV were listed from the cross-section tables for “alpha on I-127” and “alpha on Na-23” in the ACSELAM library (https://wwwndc.jaea.go.jp/ftpnd/sae/acl.html).

**Supplemental Table 2.** Decay data from nuclides produced in this study.

| Nuclide | T_1/2_ | β^+^ intensity | E_γ_ |
| --- | --- | --- | --- |
| (Desired nuclides) | |  |  |
| ^127^Cs | 6.25 h | 3.04% | 412.0 keV (62.9%) |
|  |  |  | 124.7 keV (11.4%) |
|  |  |  | 462.3 keV* (5.1%) |
|  |  |  |  |
| ^129^Cs | 32.06 h | 0.0029% | 371.9 keV* (30.6%) |
|  |  |  | 411.5 keV (22.3%) |
|  |  |  |  |
| (By-product nuclides) | |  |  |
| ^24^Na | 14.96 h | - | 1369.6 keV* (100%) |
|  |  |  | 2754.0 keV (99.9%) |
|  |  |  |  |
| ^124^I | 4.18 d | 22.40% | 602.7 keV* (62.9%) |
|  |  |  | 722.8 keV (10.4%) |
|  |  |  |  |
| ^126^I | 12.93 d | 1.01% | 388.6 keV (35.6%) |
|  |  |  | 666.3 keV* (32.9%) |

The energies with an asterisk were used for quantification.

**
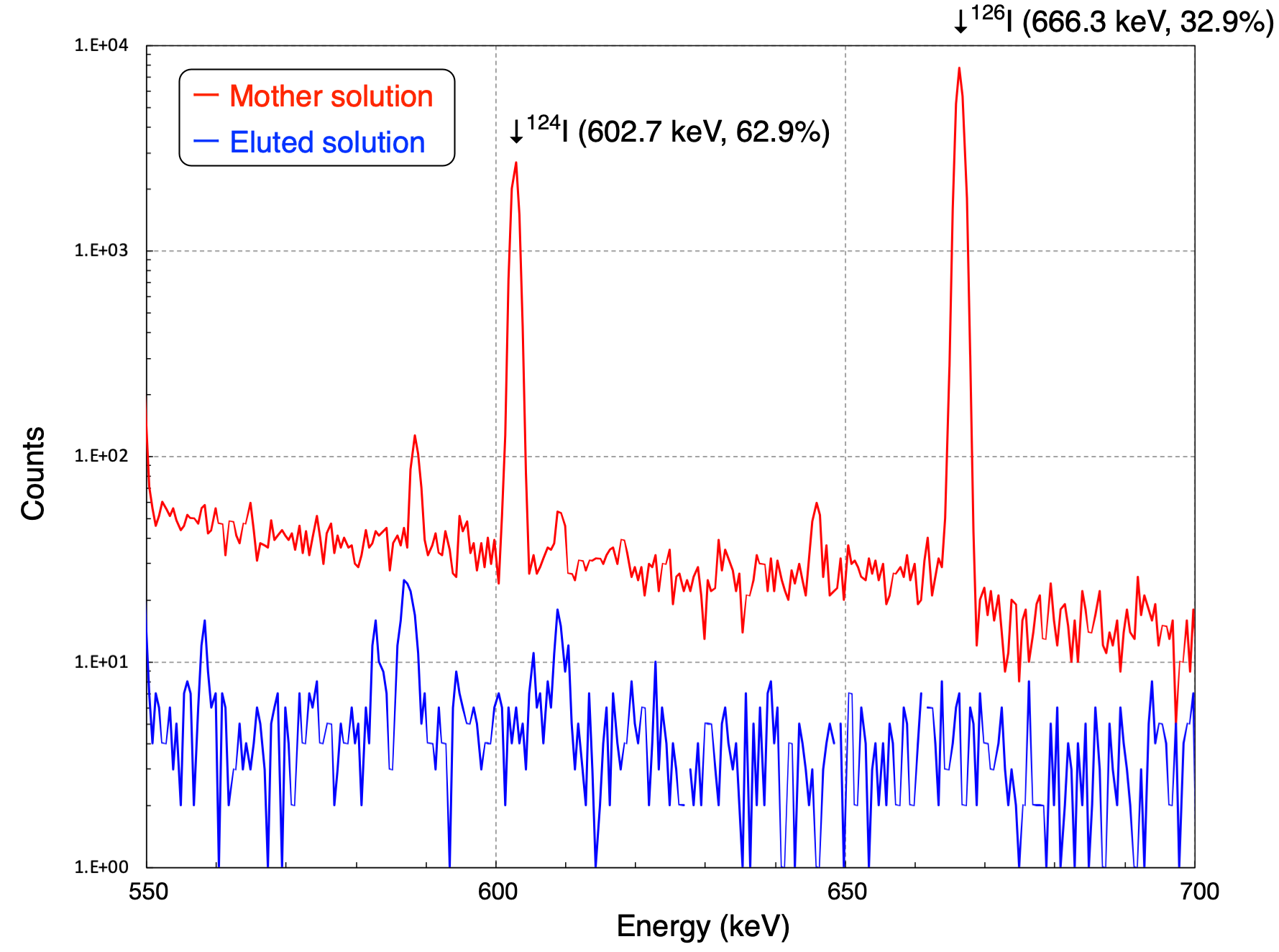
Supplemental Figure 2.** Gamma-ray spectrum of the mother and eluted solutions measured for 3 h at 3.5 cm from the detector, 7.4 days (the mother solution) and 2.4 days (eluted solution) after end of irradiation. The samples shown in Fig. 1 were measured.

**
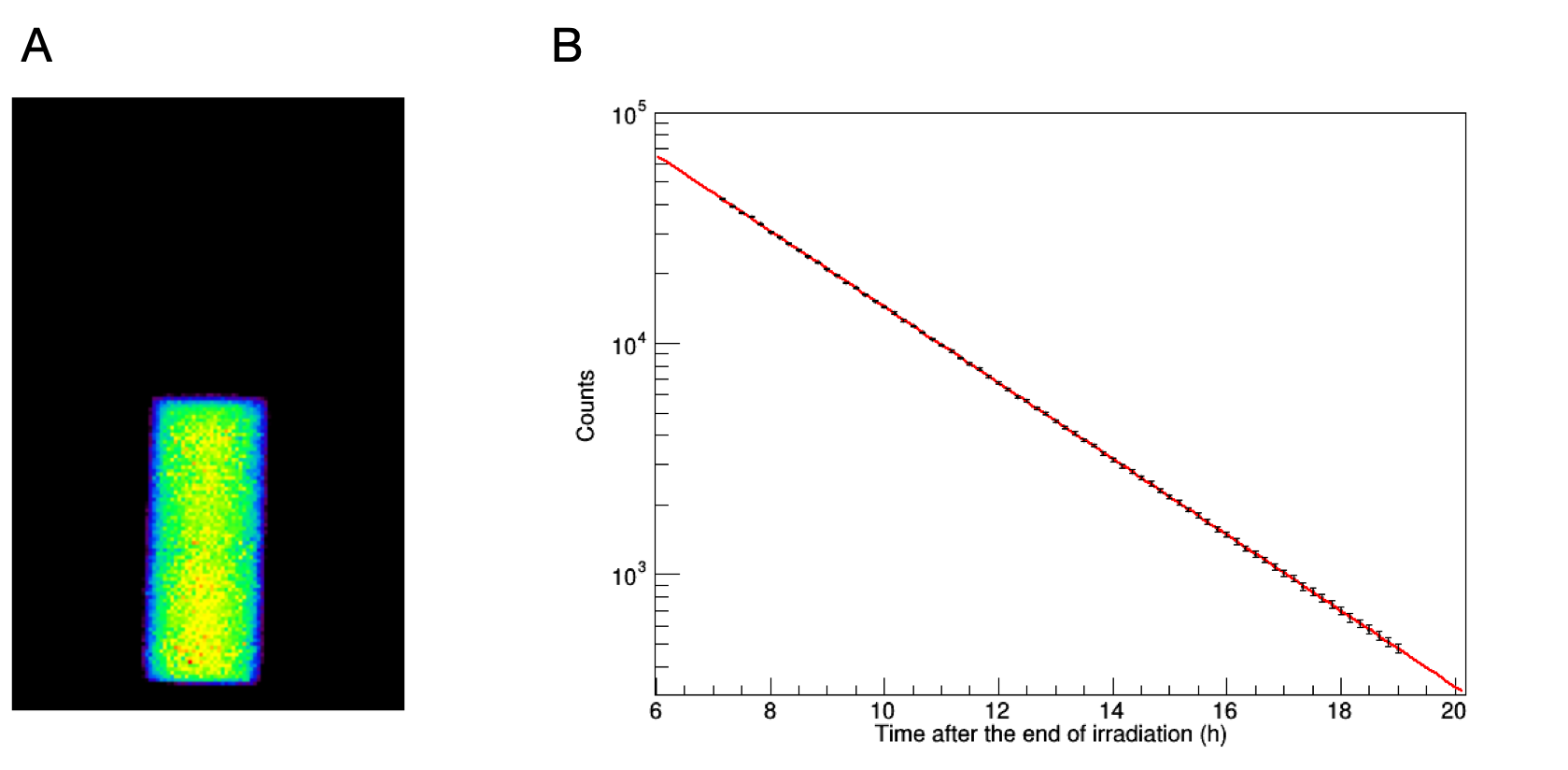
**

**Supplemental Figure 3.** (A) Twelve-hour integrated image of the effluent solution from the Cs adsorbent column obtained by the planar positron imaging system. (B) Time course plot of raw counts and estimation of half-life. The red line represents the result of one-component fitting. Error bars represent statistical errors in measurement.


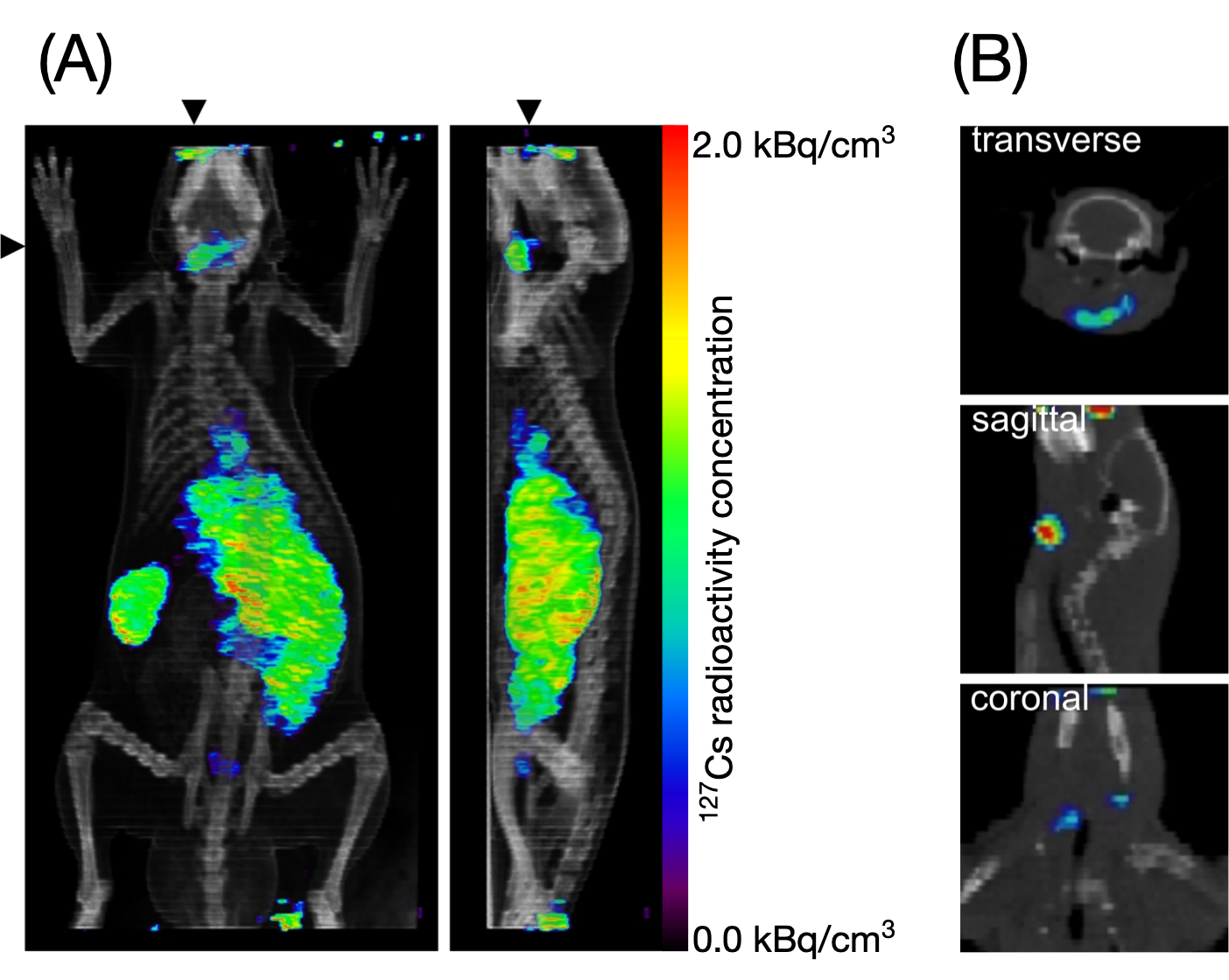


**Supplemental Figure 4.** Integrated image of PET imaging of ^127^Cs in a living rat in Experiment 1. (A) Three-hour integrated image of ^127^Cs in the whole body of the rat. The triangles indicate the location of the cross section. (B) Cross-sectional view of the integrated image of ^127^Cs in the neck.


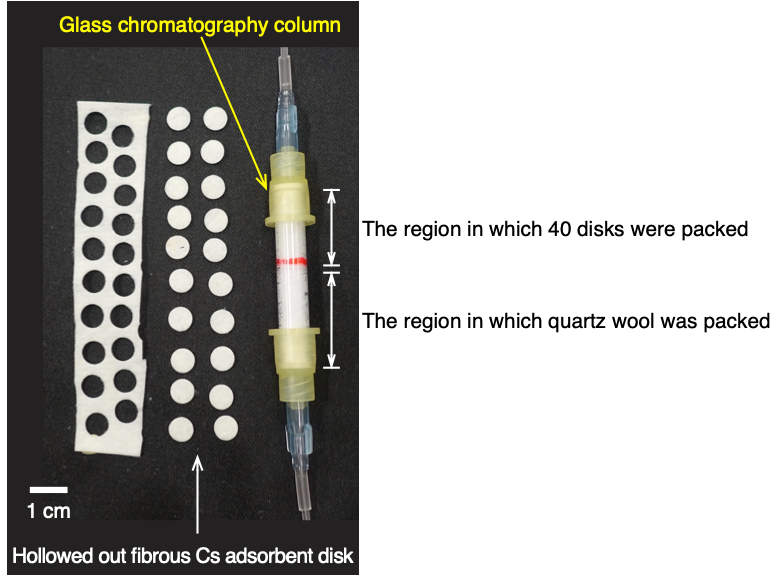


**Supplemental Figure 5.** Structure of the Cs adsorbent column.
